# A photoacoustic image reconstruction method using total variation and nonconvex optimization

**DOI:** 10.1186/1475-925X-13-117

**Published:** 2014-08-17

**Authors:** Chen Zhang, Yan Zhang, Yuanyuan Wang

**Affiliations:** Department of Electronic Engineering, Fudan University, Shanghai, 200433 China

**Keywords:** Photoacoustic imaging, Image reconstruction, Total variation, *L*_*p*_-norm, Nonconvex optimization

## Abstract

**Background:**

In photoacoustic imaging (PAI), the reduction of scanning time is a major concern for PAI in practice. A popular strategy is to reconstruct the image from the sparse-view sampling data. However, the insufficient data leads to reconstruction quality deteriorating. Therefore, it is very important to enhance the quality of the sparse-view reconstructed images.

**Method:**

In this paper, we proposed a joint total variation and *L*_*p*_-norm (TV-*L*_*p*_) based image reconstruction algorithm for PAI. In this algorithm, the reconstructed image is updated by calculating its total variation value and *L*_*p*_-norm value. Along with the iteration, an operator-splitting framework is utilized to reduce the computational cost and the Barzilai-Borwein step size selection method is adopted to obtain the faster convergence.

**Results and conclusion:**

Through the numerical simulation, the proposed algorithm is validated and compared with other widely used PAI reconstruction algorithms. It is revealed in the simulation result that the proposed algorithm may be more accurate than the other algorithms. Moreover, the computational cost, the convergence, the robustness to noises and the tunable parameters of the algorithm are all discussed respectively. We also implement the TV-*L*_*p*_ algorithm in the *in*-*vitro* experiments to verify its performance in practice. Through the numerical simulations and *in*-*vitro* experiments, it is demonstrated that the proposed algorithm enhances the quality of the reconstructed images with faster calculation speed and convergence.

## Introduction

Photoacoustic imaging (PAI), also known as optoacoustic tomography (OAT) or thermoacoustic tomography (TAT), is a novel hybrid biomedical imaging modality which combines the strengths of both optical and ultrasound imaging [1–6]. Due to its non-ionizing nature, it has been considered as a promising imaging technique and developed rapidly during the past decade. PAI reveals physiologically specific optical absorption contrast of the biological tissues, which has great potential in clinic applications such as early tumor detection [7, 8], vessel imaging [9, 10] and brain imaging [11].

PAI is developed based on the photoacoustic effect [1, 2], which is a process describing that the imaging tissues absorb the laser energy and convert it into acoustic waves. In this paper, we focus on the computed-tomographic PAI. In this kind of imaging mode, a laser pulse is used to illuminate the imaging tissues from the top. Some of the laser energy is absorbed and converted into heat, leading to thermoelastic expansion and thus wideband ultrasonic wave emission. The generated photoacoustic signals are then detected by a scanning ultrasound transducer or a transducer array to form images. Based on these detected signals, the optical absorption deposition of the imaging tissues can be calculated by using an image reconstruction algorithm.

In PAI, the reconstruction algorithms have become the vital factor of imaging quality. The PAI reconstruction result benefits a lot from a stable, accurate and efficient algorithm. A variety of analytical image reconstruction algorithms have been developed. Reconstruction algorithms based on the inverse spherical radon transform have been proposed in both the time-domain [12, 13] and the frequency-domain [14, 15]. The filtered back-projection (FBP) algorithm proposed by Xu *et al.* is the most popular algorithm due to its accuracy and convenience [16, 17]. The deconvolution reconstruction algorithm proposed by Zhang *et al*. has specific advantage under the circumstance of limited-angle sampling and heterogeneous acoustic medium [18, 19]. Several investigations have been made to propose the algorithms in plane geometries for imaging with the linear array of transducer [20, 21]. The analytical reconstruction methods have advantage in the computational cost and implementation convenience. However, the analytical algorithms fail to keep effective when the sampling points are sparse. The ignorance of measurements noises leads to the severely quality decline in the noisy situation. Those drawbacks above limit the applications of the analytical algorithms and impair their performance. Then the iterative image reconstruction methods are proposed to overcome these shortcomings and enhance the image quality of PAI.

The iterative image reconstruction methods usually build up a model to describe the relationship between the detected photoacoustic signals and the optical absorption deposition. So they are also called the model-based algorithms. Most of them calculate the optical absorption deposition iteratively to get the final reconstructed image. With proper optimization condition setup, the model-based methods can provide a more accurate and robust image reconstruction compared to the analytical ones [22, 23]. Many methods that proved to be useful in other aspects have been adopted in PAI reconstruction as an optimization condition of the model-based methods. Some algorithms focus on the compensation of the acoustic inhomogeneous phenomenon [24, 25]. Jose *et al.* proposes an iterative approach that takes the speed-of-sound of subject into account. They acquire the 2D speed-of-sound distributions and use this speed-of-sound map in their reconstruction algorithm [24]. Huang *et al.* develop and establish a full-wave iterative reconstruction approach in PAI to deal with the acoustic inhomogeneous and acoustic attenuation problem [25]. The compressed sensing has been involved in PAI reconstruction aiming to reduce the measurements and accelerate the data acquisition [26, 27]. The model-based algorithm proposed by Rosenthal *et al*. recovers the image in the wavelet domain with a different strategy [28]. Meng *et al.* develop a compressed sensing framework by using partially known support [29, 30]. The reported results show some improvement of image qualities. The total variation (TV) coefficient is always used to de-noise the image. Some algorithm are proposed by using the total variation minization to PAI image reconstruction [31–34]. Yao *et al.* propose the total variation minization (TVM) with the TV coefficient involved in the finite elment method to enhance the image quality and overcome the limit-angle problem [31, 32]. An adaptive steepest-descent-projection onto convex sets (ASD-POCS) is proposed by Wang *et al.* with the TV utilized in the iteration [33]. They investigate and employ the TV-based iterative image reconstruction algorithms in three-dimensional PAI. Zhang *et al*. utilizes the TV coefficient along with the gradient descent method in PAI reconstruction to propose the Total variation based gradient descent (TV-GD) algorithm [34]. The TV-GD method is reported to be stable and efficient under the sparse-view circumstance for PAI reconstruction. From the discussion noted above, it can be deduced that the iterative algorithms for PAI reconstruction have advantage in reconstruction qualities and robustness to noise. Now the reduction of scanning time is the main concern of PAI. A popular strategy is to reconstruct the image from sparse-view sampling data. Also there exists photoacoustic imaging system which can image the whole area with one laser exposure. These systems usually have large amount of transducers around the imaging area. With the help of sparse-view photoacoustic imaging reconstruction method, the transducer amount can be reduced. This reduction benefits the system from two main aspects. First, it helps to manage the system complexity to a lower level. The lower complexity system is more stable and easier to maintain. Second, this reduction also means the reduction of data scale. The data scale reduction can make the acquisition process more simple and flexible. Besides these two aspects, it is also worth to mention that it reduces the cost of the whole system. All those mentioned above is very important for further clinical applications. It is very important to develop a sparse-view imaging system. In this situation, the qualities of the iterative reconstructed images have room for improvement. Take the TV-GD method for example, it is reported to be an efficient and high-quality algorithm in sparse-view situation. But the paintinglike artifacts emerge and some detail information is lost in the extremely sparse-view reconstruction.

The compressed sensing theories have been adopted in PAI reconstruction, in which the *L*_1_-norm of the signal is minimized to obtain the reconstructed image. Recently, Chartrand reported that by replacing the *L*_1_-norm with the *L*_*p*_-norm (0 < *p* ≤ 1), accurate reconstruction is possible with substantially fewer measurements [35]. This nonconvex optimization setting has been successfully applied to Magnetic Resonance Imaging (MRI) image reconstruction [36, 37]. The results show that the algorithm with *L*_*p*_-norm can provide accurate reconstruction image with fewer measurements comparing to the *L*_1_-norm based algorithms. To another dimension of this optimization problem, several algorithms have been proposed to get a better performance in image reconstruction through jointly minimizes the TV value and *L*_1_-norm value [38, 39] in MRI image reconstruction.

In this paper, we present a novel algorithm to the problem of reconstructing the image from sparse-view data in PAI. The algorithm is based on the jointly minimization of total variation and nonconvex *L*_*p*_-norm (TV-*L*_*p*_). The reconstructed image is updated by calculating its joint total variation value and *L*_*p*_-norm value. The operator-splitting framework is used to reduce the computational cost, and the Barzilai-Borwein step size selection method is adopted to obtain the faster convergence. Through the numerical simulation, the image reconstruction in the case of insufficient sampling data was accomplished. The reconstruction result is compared with several other algorithms including the FBP [16], the *L*_1_-norm [27] and the TV-GD method [34]. The computational cost and the convergence of the proposed algorithm is also discussed and compared with other algorithms. The numerical simulations also cover the robustness to the noise and the tunable parameter investigation. Through the numerical simulations and *in*-*vitro* experiments, it is demonstrated that the proposed algorithm enhances the quality of the reconstructed images with the faster calculation speed and convergence. It’s worthwhile to mention that like Ref. [27] and other iteration method, we also used a projection matrix to connect the acoustic pressure measurements with the reconstructed image. But there are some implementation differences between our method and that one. We both use an intermediate variable to simplify our equations. Ref. [27] used the velocity potential as the intermediate variable and we used a linear integration of the initial pressure along an arc whose center is the position of the ultrasound sensor and with a certain radius *ct*. The Ref. [27] used a sparsifying matrix and minimized the *L*_1_-norm in sparsifying domain to get the reconstruction. We used the information from sparsifying domain and piecewise continuous behavior to reconstruct the image. Also, we adapted the *p*-norm minimization into the algorithm, so it can be a more accurate algorithm in sparse-view PAI.

The main contribution of this paper is to develop a novel algorithm for solving the problem of reconstructing the image from sparse-view data in PAI. Our contributions are threefold. First, we include the nonconvex optimization into the PAI reconstruction. This nonconvex optimization setting can provide more stable and accurate result under the sparse-view situation. Second, we combine the nonconvex optimization with TV minimization. The combined method is able to reconstruct more detailed image with sharp edge. Finally, we implement the Barzilai-Borwein method accelerates the reconstruction speed and improves the convergence considerably.

This paper is organized as follows. ‘Theory and method’ describes the theory of the proposed algorithm. The numerical simulation is introduced in ‘Simulation’. The *in*-*vitro* experimental results are shown in ‘*In-vitro* experiments’. The conclusions of this work are drawn in ‘Conclusion’.

## Theory and method

### Photoacoustic theory

In this paper, the two-dimensional PAI is concerned in the simulations and experiments. In 2D PAI, a laser pulse is used to illuminate the imaging tissues from the top. Due to the photoacoustic, the illumination creates an initial acoustical pressure field. The initial acoustical pressure field propagates as ultrasound waves, which can be detected by ultrasound transducers. Based on the physical principle of the photoacoustic effect, assuming that the illumination is spatially uniform, the relationship between the acoustical pressure measurements and the initial pressure rise distribution can be derived as :
1

where  is the acoustic pressure measurements at the position r and the time *t*, *c* is the sound speed, *C*_*p*_ is the specific heat, μ is the isobaric expansion coefficient, *I*(*t*) is the temporal profile of the laser pulse and  is the initial pressure rise distribution. In our study and many photoacoustic tomography studies, we employ a laser pulse with a very short duration. Its duration is nano seconds. So here we made an approximation to treat the *I*(*t*) as a Dirac-delta function.

In order to recover initial data for the wave equation, several inversion formulas have been established to solve this as a filtered back-projection problem [12, 40]. By using the Green’s function [12] to solve equation (1), the acoustic pressure measurements can be deduced as:
2

where  is the position of the ultrasound transducer.

In PAI experiments, an ultrasound transducer is used to receive the acoustic pressure measurements at different positions, and the image reconstruction is regarded as an inverse problem to obtain the initial pressure rise distribution. In the iteration of the image reconstruction, a projection matrix *A* is typically established to connect the acoustic pressure measurements with the reconstructed image. The measurements can be calculated based on the reconstructed image, and then the reconstructed image can be repeatedly corrected by minimizing the difference between the calculated measurements and the real ones. In this way, the optimization method can be used for collaboration and then the iteration reconstruction algorithm can be developed.

### Compressed sensing for PAI

If the sampling data is insufficient, the projection matrix *A* is ill-conditioned. Thus, the matrix *A* does not have an exact inversion. As a result, it leads to streaking artifacts in the reconstructed image. This problem can be treated by incorporating the compressed sensing theory into PAI.

We define a new variable *f* as:
3

Then the equation (2) can be converted as follows:
4

In practical imaging, the reconstructed image and the measurements are processed discretely, and the image is reshaped into vectors for convenience. If the size of the reconstructed image  is *X* pixels × *Y* pixels, then the total pixel number of the reconstructed image  is *N* (*N* = *XY*). After vectorization, the reconstructed image  becomes a vector u with the length of *N*. If the total number of the detection points is *Q*, the length of measurement in each detection point is *M*, the equation (4) can be expressed as:
5

where *f*_*i*_ is the integration of the  along the arc that is centered in *i*th detection point and with a radius of *ct*, *A*_*i*_ is the projection matrix of the *i*th detection point, T is the transpose operation of a matrix. The calculation of the projection matrix is as follows:
6

- Calculate an matrix *A*_*i*_(*j*) as:

where  , (*m*, *n*) is the position of the *j*th point in the reconstructed image,(*m*_*i*_, *n*_*i*_) is the position of the *i*th detection point,d*x* is the actual length between the two pixels in the reconstructed image,d*t* is the discretized time step and *M* is the total sampling points at one detection point.
7

(b) Vectorize the matrix *A*_*i*_(*j*) as the *j*th column vector in projection matrix *A*_*i*_.
(c) Repeat the calculation *M* times to get the projection matrix *A*_*i*_.
(d) Repeat step(a) to step(c) *Q* times to get the projection matrix in the different sampling positions (*A*_1_,*A*_2_,…,*A*_*Q*_). Then write the projection matrixes in the forms as follows:

The equation (6) can be expressed as:
8

where the sizes of *f*, *A* and u are *MQ* pixels × 1 pixel, *MQ* pixels × *N* pixels and *N* pixels × 1 pixel respectively.

To reconstruct the photoacoustic image from incomplete measurements by using the compressed sensing theory, we can solve an optimization problem as follows:
9

where Ψ is a sparse transform matrix,| |_1_ and | |_2_ are the *L*_1_-norm and L_2_-norm respectively. By projecting the image onto an appropriate basis set, we can get a sparse representation of the original image. In this domain, most coefficients of the image are small, and a few large coefficients capture most information of the signal. In this way, we can recover a much more accurate image from those undersampled measurements.

In practical applications of PAI, the reconstructed images often show piecewise continuous behavior. The images like this always have small total variation (TV) values, which is defined as follows:
10

where *D*_*i*_ is a matrix with the size of 2 pixels × *N* pixels that has two nonzero entries in each row to calculate the finite difference of *u* at the *i*th pixel. *D* is a matrix with the size of 2*N* pixels × *N* pixels, and *D =* (*D*^*X*^;*D*^*Y*^),*D*^*X*^ and *D*^*Y*^ are the horizontal and vertical global finite difference matrixes respectively.

It is reported that the TV based reconstruction algorithm can recover the image accurately from sparse sampling data [34]. Using TV values to reconstruct the image can be expressed mathematically as:
11

However, the TV minimization still has some limitations that impair its performance. The optimization of the TV value encourages the recovery of images with sparse gradients, thus resulting in the paintinglike staircase artifacts in the reconstructed images.

Recently, some research find out that the nonconvex optimization can reconstruct an accurate image with fewer measurements by replacing the *L*_1_-norm with the *L*_*p*_-norm (0 < *p* ≤ 1). Aiming to enhance the reconstruction quality and overcome the problem of TV based algorithm, we joint the *L*_*p*_-norm with TV values to establish a new optimization which can be defined by:
12

where α and β are parameters corresponding to the weights of the TV value and *L*_*p*_-norm value , | |_*p*_ is the *L*_*p*_*–*norm in this optimization problem respectively.

Therefore, we can obtain the reconstructed image by solving this new optimization problem in equation (12).

### PAI reconstruction algorithm

In this part, we solve the optimization problem in equation (12) to establish a novel photoacoustic image reconstruction algorithm by using the total variation and nonconvex optimization.

We define the finite difference approximations to partial derivatives of u at the *i*th pixel along the coordinate as variable *ω*_*i*_ = *D*_*i*_u, the *i*th pixel’s sparse coefficient as variable z_*i*_ = *Ψ*_*i*_^T^u, where Ψ_*i*_ is the sparse transform matrix of the *i*th pixel. The equation (12) can be deduced as:
13

where ρ is the parameter corresponding to the weight of the constraint condition in this optimization problem.

We form the augemented Lagrangian defined by
14

where *b*_*i*_^*k*^ is the TV step parameter in *k*th iteration, *c*_*i*_^*k*^ is the *L*_*p*_-norm step parameter in *k*th iteration, u^k^ is the vectorized image reconstruction in *k*th iteration.

This problem can be solved by
15

where ω^*k*+1^ is the finite difference approximations to partial derivatives of u in (*k* + 1)th iteration, *z*^*k*+1^ is the sparse coefficient in (*k* + 1)th iteration, u^*k*+1^ is the vectorized image reconstruction in (*k* + 1)th iteration, *z*_*i*_^*k*+1^ is the sparse coefficient of the *i*th pixel in (*k* + 1)th iteration, ω_*i*_^*k*+1^ is the finite difference approximations to partial derivatives of u at the *i*th pixel along the coordinate in (*k* + 1)th iteration; *b*_*i*_^*k*+1^ is the TV step parameter in (*k* + 1)th iteration, *c*_*i*_^*k*+1^ is the *L*_*p*_-norm step parameter in (*k* + 1)th iteration.

By using the standard augmented Lagrangian method, the optimization problem in (15) can be deduced as
16

where ω^*k*^ is the finite difference approximations to partial derivatives of u in *k*th iteration, *z*^*k*^ is the sparse coefficient in *k*th iteration, u^*k*^ is the vectorized image reconstruction in *k*th iteration; δ_*k*_ is the Barzilai-Borwein step parameter in *k*th iteration.

After using the Barzilai-Borwein method to determine the step size δ, the optimization problem in equation (13) can be transformed into three sub-problem as follows:
17

where ω_*i*_^*k*^ is the finite difference approximations to partial derivatives of u at the *i*th pixel along the coordinate in *k*th iteration respectively, *z*_*i*_^*k*^ is the sparse coefficient of the *i*th pixel in *k*th iteration respectively; δ_*k*+1_ is the Barzilai-Borwein step parameter in (*k* + 1)th iteration.

We use the soft shrinkage operator to obtain the solution to ω-subproblem in equation (17), the operation is as follows:
18

where *a*_1_, *a*_2_, *t*_1_ and *t*_2_ are the variables used for a succinct expression.

As for the *z*-subproblem in equation (17), we use the soft *p*-shrinkage operator to solve it. The operator is defined by:
19

where *a*_3_, *a*_4_, *t*_3_ and *t*_4_ are the variables used for a succinct expression.

The *u*-subproblem in equation (17) is a typical least squares problem. The solution can be easily obtained by:
20

where *F* is the Fourier transform matrix.

As a result, the **TV-*L***_***p***_ algorithm is summarized as follows:
21

1. Initialization: input *f,* α, β, ϵ *,p* and ρ. Set the reconstructed image u^0^ = 0, *b = c* = 0, δ_0_ = 1, *k* = 0.
2. Apply equation (18) and (19) to update the value of ω and *z*.
3. Apply equation (20) to update the value of u.
4. Apply equation (17) to update the value of *b*, *c* and δ.
5. If the exiting condition is met, end the iterations and output the result. Otherwise repeat the step from (2) to (4). The exiting condition is as follows:

## Simulation

To verify the effectiveness of the proposed TV- *L*_*p*_ algorithm on PAI reconstructions, the simulations are designed. All the simulations are performed in Matlab v7.14 on a PC with a 3.07 GHz Intel Xeon processor (only 1 core is used in computation) and 32 GB memory. The sparsisfying operator Ψ is set to Haar wavelet transform using Rice wavelet toolbox. The sound speed is set to be consistent in the simulation as 1500 m/s.

### Sparse-view reconstruction

**Figure 1:**
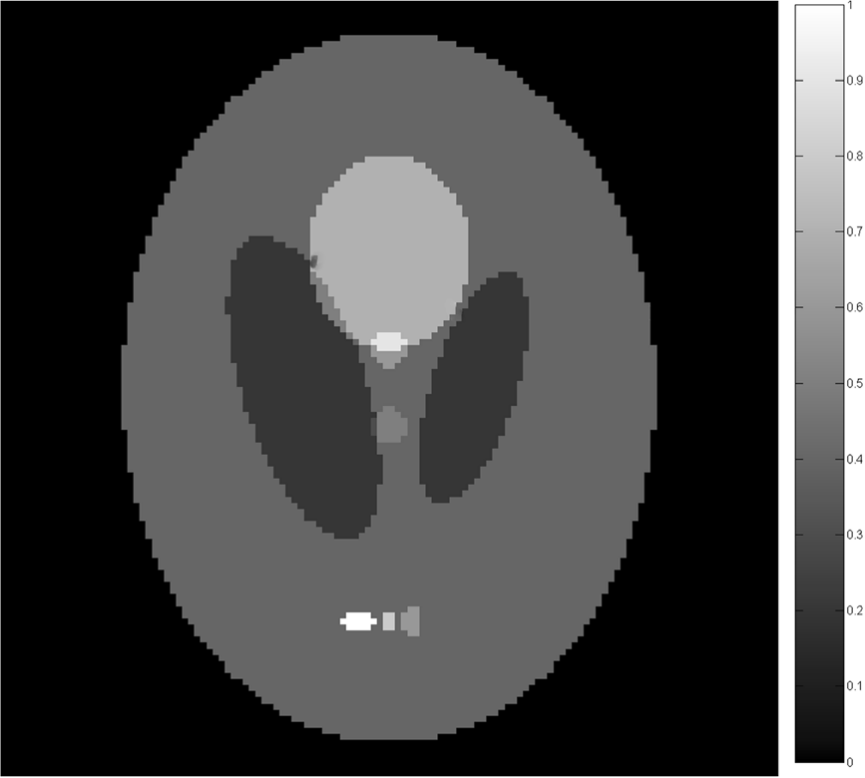
**The shepp-logan phantom.**

In the simulation, we choose the Shepp-Logan phantom to be the initial pressure rise distribution. The forward simulation and inverse reconstruction are all performed in 2D. The phantom is shown in Figure 1. The measurements from the phantom are generated by using equation (2). The size of the phantom is 89.6 mm × 89.6 mm, the radius of the scanning circle is 42 mm and the size of the reconstructed image is 128 pixels × 128 pixels. During the simulation, the scanning circle covers 360°around the imaging phantom. Four different measurements are collected. The scanning step of tomographic angels is set to 2.25°, 4°, 12° and 20° respectively. So the sampling points are 160 views, 90 views, 30 views and 18 views correspondingly.

The parameters α, β, ϵ and ρ are set to be 1 × 10^-2^, 1 × 10^-2^, 1 × 10^-5^ and 1 respectively. The influence of these parameters will be discussed later. And the parameter *p* are set to be two different values as 0.5 and 0.8.

**Figure 2:**
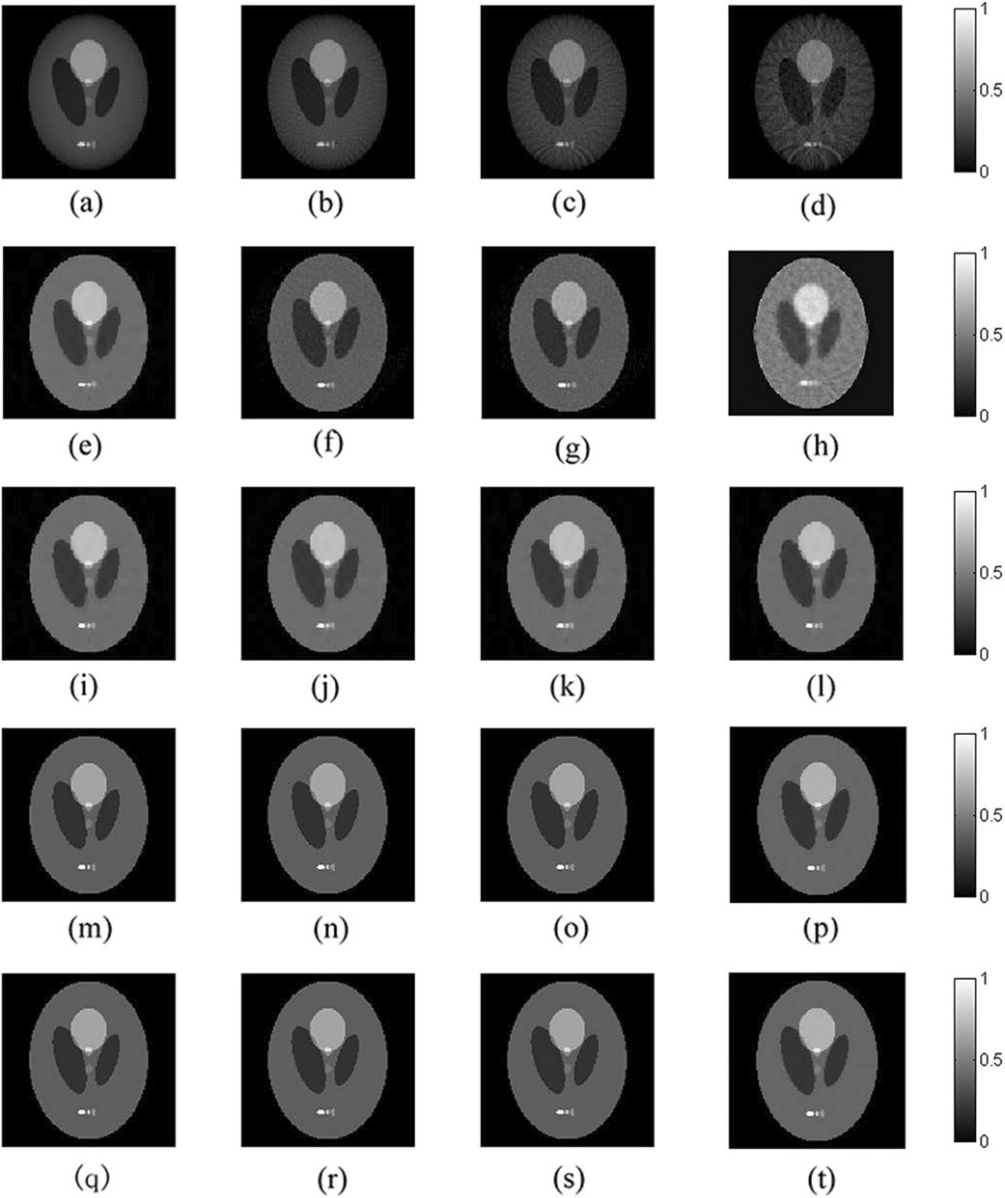
**The shepp-logan phantom reconstruction results by different algorithms.** The first to fifth rows refer to the FBP **(a-d)**, L1-norm **(e-h)**, TV-GD **(i-l)**, TV-Lp (p = 0.5) **(m-p)** and TV-Lp (p = 0.8) **(q-t)** reconstructed images respectively and the first to fourth columns refer to the results from 160-view, 90-view, 30-view and 18-view respectively.

We choose the FBP [16], the *L*_1_-norm [27] and the TV-GD [34] algorithms to be the comparison besides our proposed TV- *L*_*p*_ algorithm. The simulation results by using these different algorithms are shown in Figure 2. It’s worthwhile to note that the weight used in the TV-GD algorithm is an adaptive parameter, as same as it is reported in [27]. The negative values in FBP reconstructed image are set to be zero.

It is shown in the first column of Figure 2 that, all three iterative algorithms have comparable reconstruction results when the sampling data is sufficient. Moreover, it is shown in Figure 2(a) that the contrast of FBP reconstructed image is not as high as the other three. But its resolution is comparable with the others visually. When the number of sampling points reduces, the qualities of the reconstructed images are strongly affected in the FBP reconstruction. When the sampling point gets sparse in the FBP reconstruction, the arc-like artifacts appears due to the back-projection arcs cannot be canceled out with each other. The iterative algorithms can provide better qualities of the reconstructed images than the FBP method in sparse-view reconstructions. Among them, the *L*_1_-norm method struggles to depress the noise. Meanwhile, the TV-GD algorithm and the TV-*L*_*p*_ algorithm provides high-resolution images and have no visually distinguishable decline in qualities of the reconstructed images as the number of sampling points decreases.

As for the extreme sparse sampling points situation (18-view and 30-view), the image reconstructed by the FBP algorithm, shown in Figure 2(d), has extremely severe artifacts. The *L*_1_-norm reconstruction and the TV-GD algorithm have a decline in image qualities. The noise in the reconstructed images by the *L*_1_-norm reconstruction, as shown in Figure 2(j), cannot be depressed effectively. As for the TV-GD algorithm, the reconstruction produces piecewise artifacts which also make the qualities of the reconstructed images decrease. In the TV-*L*_*p*_ image, the noise is depressed more effectively than the above three algorithm. The quality of the reconstructed image is not affected substantially by the insufficient sampling data.

We calculate the peak signal-to-noise ratios (PSNR) of the reconstructed images with the original phantom as a gold standard to provide a numeric quantification of the results. The bigger the value of PSNR is, the better quality of the image is. The PSNR is defined as:
22

where *t*(*i,j*) means the gray-value of the original image, *MAX* the maximum possible pixel value of the image which in our simulation is 1.

We calculate the value of the PSNR of all images in Figure 2. The quantitative results are shown in Table 1.

**Table 1:** **PSNRs (dB) of reconstructed images of shep-logan phantom**

	160-view	90-view	30-view	18-view
FBP	20.35	18.36	15.68	13.14
*L*_1_-norm	33.83	34.98	34.21	31.19
TV-GD	38.01	38.23	36.68	34.68
TV-*L*_*p*_(*p* = 0.8)	38.45	39.05	36.91	36.72
TV-*L*_*p*_(*p* = 0.5)	38.85	39.27	37.01	36.81

From Table 1, it is shown that the PSNR of the FBP algorithm is always in a very low level due to its unsuitability for sparse-view sampling condition. As for those three compressed sensing based algorithms, the PSNR value of images reconstructed by the TV-*L*_*p*_ algorithm are the highest. The *L*_*p*_*-*norm optimization constraint can provide the better performance in the extremely sparse sampling. With this improvement, the TV-*L*_*p*_ algorithm is more accurate than other algorithms in the sparse-view sampling condition shown in the quantitative results. Between the two different value of *p*, the parameter *p* that is set to be 0.5 has a slightly advantage against the other one. Also it is revealed from Table.1 that the 90-view shows higher PSNR than 160-view. When the sampling points are sufficient, it is possible that the fewer-view projection can produce better reconstruction results. But their PSNR is very close with same algorithm. It is fair to say that the results are on the same level of image quality.

**Figure 3:**
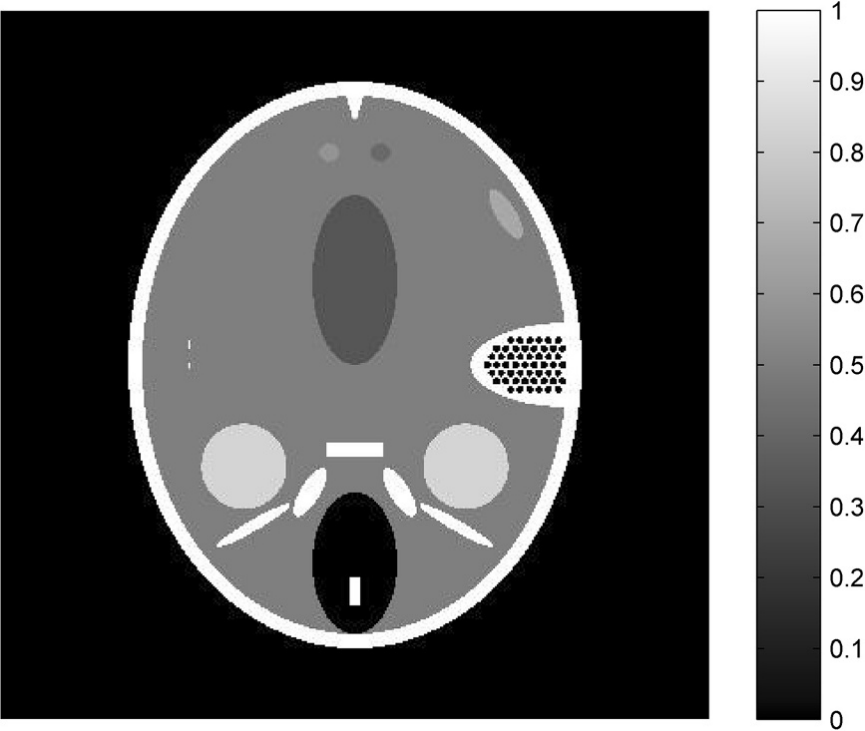
**The FORBILD phantom.**

**Figure 4:**
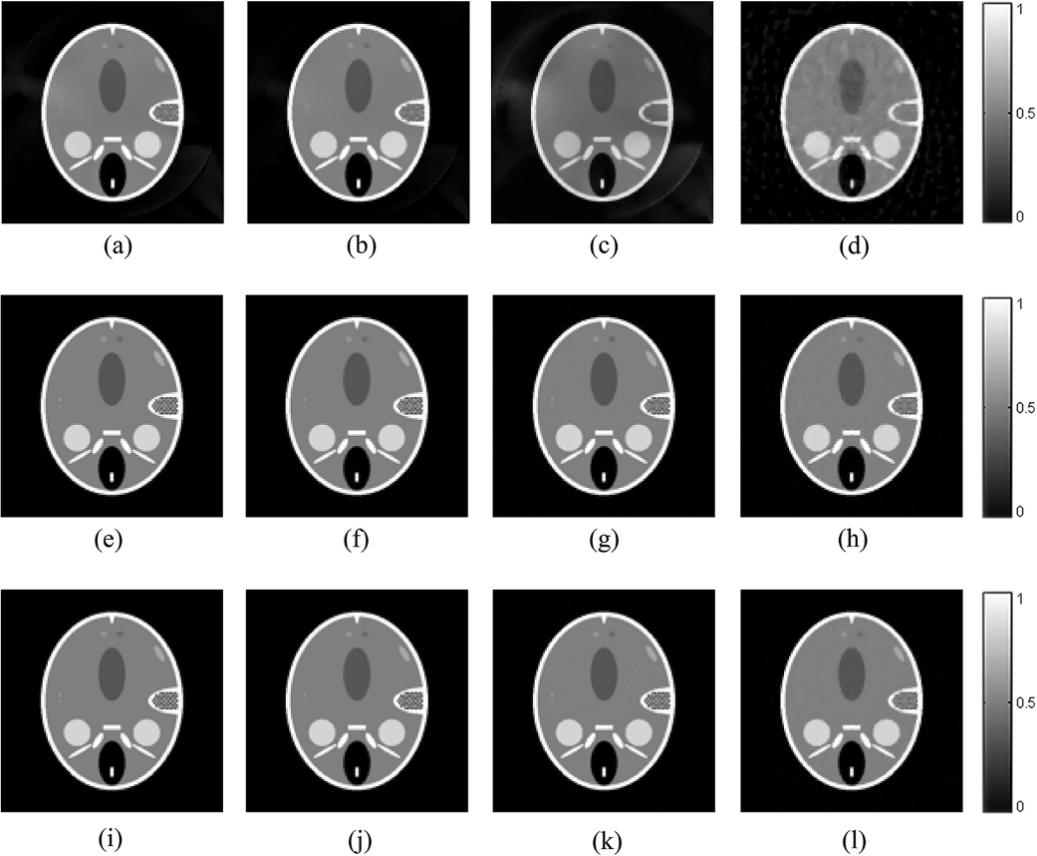
**The FORBILD phantom reconstruction results by different algorithms.** The first to the third rows show images reconstructed by the TV-GD **(a-d)** and TV-Lp algorithm with p = 0.8 **(e-h)** and p = 0.5 **(i-l)**, respectively. The first to fourth columns refer to the results from 160-view, 90-view, 60-view, 30-view respectively.

From Figure 2, it is shown that the TV-GD image and the TV-*L*_*p*_ image are very close in the image quality. Here we choose the FORBILD phantom [41], a more complicated and more challenging phantom, to further compare the proposed algorithm with the TV-GD algorithms. The phantom is shown in Figure 3. The scanning step of tomographic angels is set to 2.25°, 4°, 6° and 12°. So the sampling points are 160 views, 90 views, 60 views and 30 views correspondingly. The other numerical implementation conditions remain the same with the shep-logan simulation. The simulation results by using the TV-GD algorithms and the proposed TV- *L*_*p*_ algorithm are shown in Figure 4. It is shown in Figure 4 that, when the sampling data is sufficient, both algorithms can reconstruct the accurate image. When the sampling angles get sparse during the simulation, it is seen that the TV-GD reconstruction results in paintinglike staircase artifacts in the smooth regions. Also it fails to give the accurate image in the low contrast regions in the top and left of the phantom. The proposed algorithm provides reasonably good reconstructions in these regions. The PSNR of the reconstructed images are shown in Table 2. From this table, we observe that the TV-*L*_*p*_ algorithm provides the better PSNR for all the cases. In the case of more complicated phantom, the TV-*L*_*p*_ algorithm shows significant improvement than the TV-GD algorithm.

**Table 2:** **PSNRs (dB) of reconstructed images of FORBILD phantom**

	160-view	90-view	60-view	30-view
TV-GD	35.36	31.75	29.68	26.63
TV-*L*_*p*_(*p* = 0.8)	39.55	40.67	38.06	36.72
TV-*L*_*p*_(*p* = 0.5)	39.13	41.12	38.91	37.41

**Figure 5:**
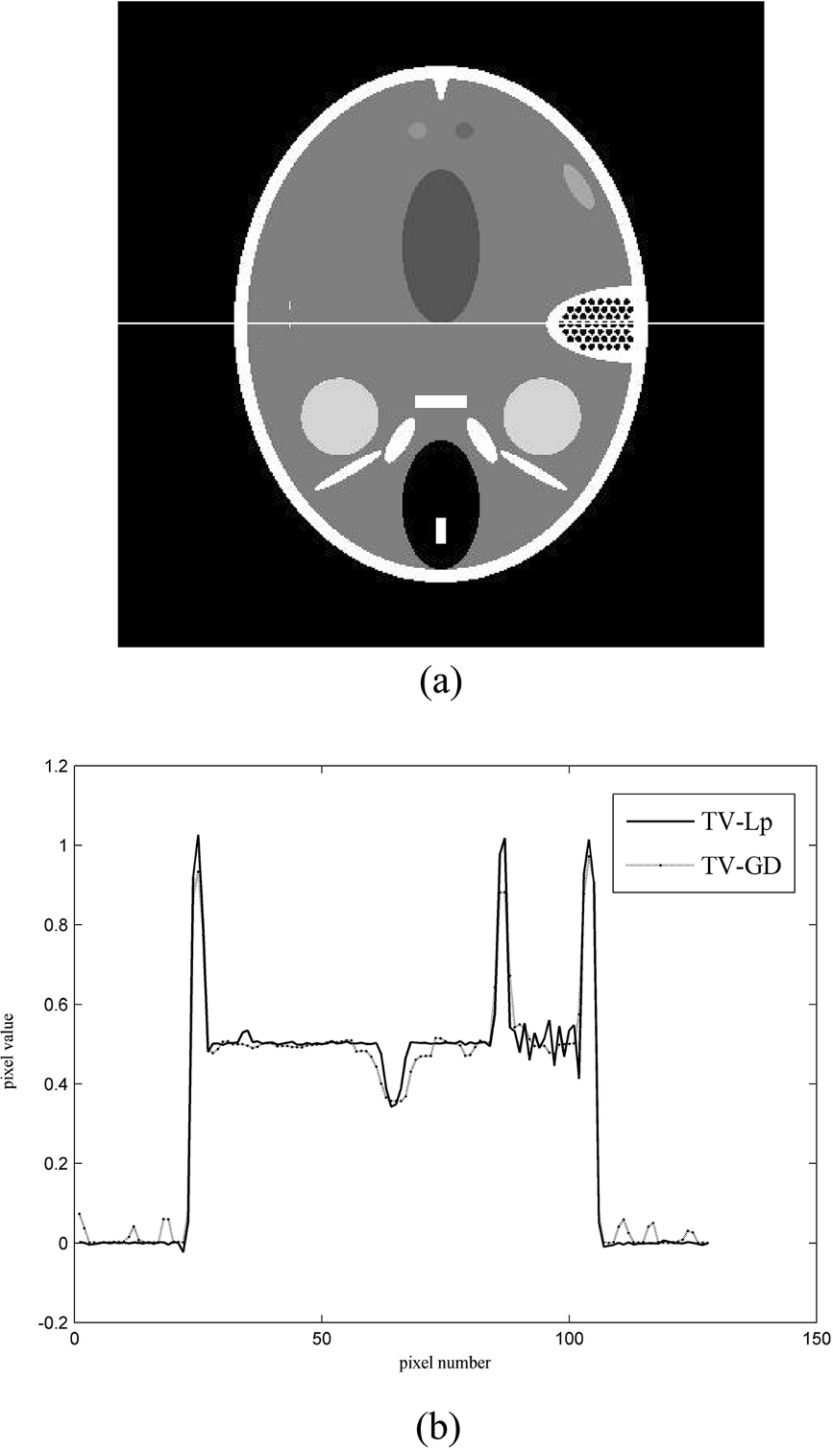
**The gray-scale profiles. (a)** The location of the pixel profile in the image. **(b)**The gray-scale profiles of the reconstructed images from 30-view data.

Also, we include a line-plots image of the reconstruction result by the TV-GD algorithm and the TV-*L*_*p*_(*p* = 0.8) algorithm from 30-view data. The location of the pixel profile in the image is displayed in Figure 5(a). The comparisons of pixel profiles are displayed in the Figure 5(b).

In Figure 5(b), the solid line and the dotted line represent the pixel profiles of the TV-*L*_*p*_ and the TV-GD image respectively. It is shown in Figure 5(b) that the TV-*L*_*p*_ can reconstruct the image more precisely than the TV-GD one. The edges from the TV-*L*_*p*_ are sharper than that from the TV-GD. The pixel number from 90 to 100 is the high resolution area, the TV-*L*_*p*_ image shows the high-speed change of the pixel value while the TV-GD fails to do so. In the continuous area, the TV-*L*_*p*_ image is smoother.

We continuously decrease the number of the detect points try to find the limit density of the sampling points. During the simulation, we set the criterion of acceptability that the PSNR of the reconstructed image reaches 30 dB. It is found out that the total number of the sampling points is able to be reduced to 15 for TV-*L*_*p*_ algorithm in the reconstruction of shep-logan phantom and to 18 for the forbild phantom.

In this part, the TV-*L*_*p*_ algorithm is proved to be more accurate and stable than the other algorithms for PAI image reconstruction in the sparse sampling condition.

### Convergence and calculation

In this part, we discuss the theoretical calculation complexity and study the convergence of the proposed algorithm. As mentioned above in ‘Theory and method’, in step (2) the update of ω and *z* is using the soft shrinkages and the computational costs are both *O*(*N*). The update of *z* also includes a wavelet transforms which computational costs are *O*(*N*log*N*). In step (3), the update of u involves two fast Fourier transforms which computational costs are *O*(*N*log*N*) and two operations of *A* with the computational costs of *O*(*NMQ*). The update of the parameters *b* and *c* in step (4) are all simple calculations with the computational costs of *O*(*N*). As for the paremeter δ, although it involves an operation of *A*, it can use the result computed in the step (3). So its computational cost is also *O*(*N*).

In a nutshell, the calculation complexity of the proposed algorithm in one iteration is 5O(*N*) + 4O(*N*log*N*) + 2O(*NMQ*). The first two terms is much smaller than the last term in the practical use of photoacoustic imaging and most iterative algorithm is with the operation. In each iteration, we just use the projection matrix twice. So the proposed algorithm has a cheap per iteration computation.

The TV-GD algorithm is reported as an efficient and stable iterative algorithm in photoacoustic imaging. In the ‘Sparse-view reconstruction’, its reconstruction result is closest to the proposed algorithm. So here we select it to be a comparison with the TV-*L*_*p*_ algorithm. We calculate the time cost of those two algorithms in a simulation. The simulation condition is same as in ‘Sparse-view reconstruction’. But the iteration ends when the PSNR values reach 30 dB. The result is shown in Table 3. From Table 3, it is shown that the proposed algorithm is faster than TV-GD algorithm in the computational time. Based on this result, it could be inferred that the TV-*L*_*p*_ algorithm is a more efficient image reconstruction algorithm comparing to the TV-GD algorithm. The value of *p* has also some influence on the time cost. The smaller the *p* is, the more iteration times are needed to reach the reconstruction result.

**Table 3:** **Time cost(second) of reconstructed images**

	160-view	90-view	30-view	18-view
TV-GD	41.79	37.19	24.63	14.61
TV-*L*_*p*_ (*p* = 0.8)	18.45	12.05	6.29	4.41
TV-*L*_*p*_ (*p* = 0.5)	20.76	14.01	8.69	6.16

Thanks to the use of Barzilai-Borwein step size selection method, the convergence speed can also be significantly improved. For the quantitative analysis, we use a parameter that represents the distance between the reconstructed image and the original phantom image. The parameter *d* is defined as:
23

where *u* is the reconstructed image and *t* is the original image. The size of the image is *X* × *Y*. The smaller the parameter *d* is, the closer the reconstructed image is with the original phantom. In the TV-*L*_*p*_ algorithm, there is a small rate of chance that the optimization will lead to the wrong solution due to its non-convex nature. So we use the original image to calculate the parameter *d* to show the image quality and use the parameter *d* as a reference. We want to show the improvement of the image quality in every iteration step.

**Figure 6:**
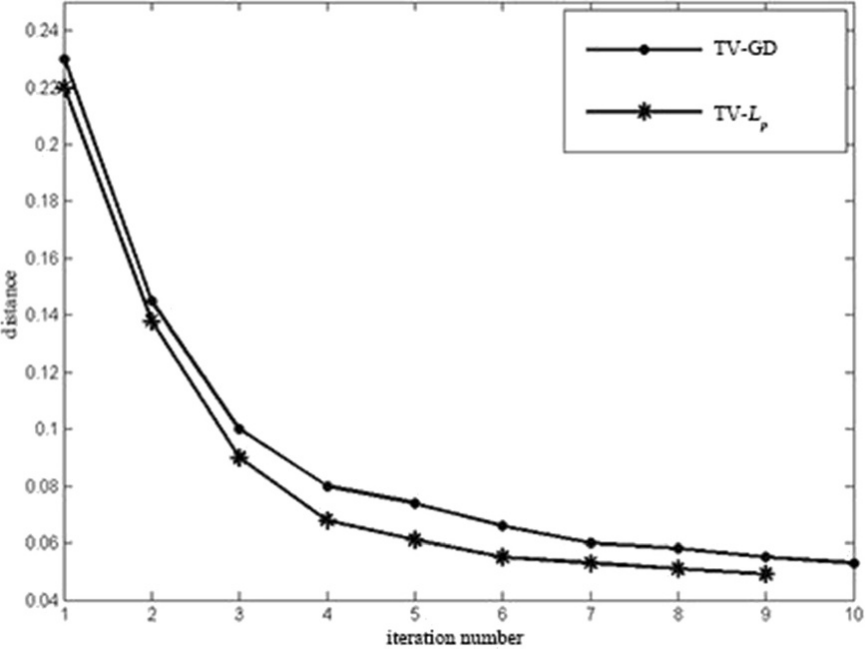
**The distance between the reconstructed images and the original phantom image versus the iteration number.**

The simulation condition is set to be the same as in ‘Sparse-view reconstruction’. The sampling view is 60. The parameter *p* is set to be 0.8. The defined distance *d* is calculated after each iteration step. If the distance is smaller than 0.05, the iteration will stop. The simulation result is shown in Figure 6. The *x*-axis is the value of distance and the *y*-axis is the iteration times. The line ‘·-’ refers to the TV-GD algorithm and the line ‘*-’ represent the TV-*L*_*p*_ (*p* = 0.8)algorithm. The result is shown in Figure 6. The images reconstructed by TV-*L*_*p*_ algorithm in each iteration have smaller value of *d* than the TV-GD ones and the TV-*L*_*p*_ iteration only takes 9 times as the distance is met the request.

For discussions noted above, it can be surmised that the convergence of TV-*L*_*p*_ algorithm is faster and the TV-*L*_*p*_ has a cheaper computational cost.

### Robustness to the noise

In the practical applications of the photoacoustic tomography, the measurements are usually polluted by those white measuring noises from the ultrasound transducer and the system electronics. Hence, it is very important for an algorithm to maintain stable performance under the noises polluted circumstances. To analyze the robustness of the TV-*L*_*p*_ algorithm, we choose 30-view simulated photoacoustic signals that we used in ‘Sparse-view reconstruction’. The signals are added with white noises of different noise power levels. We use TV-*L*_*p*_ algorithm with two different settings of parameter *p* (*p* = 0.5 and *p* = 0.8) and TV-GD algorithm to reconstruct images from these white noise polluted measurements.

**Figure 7:**
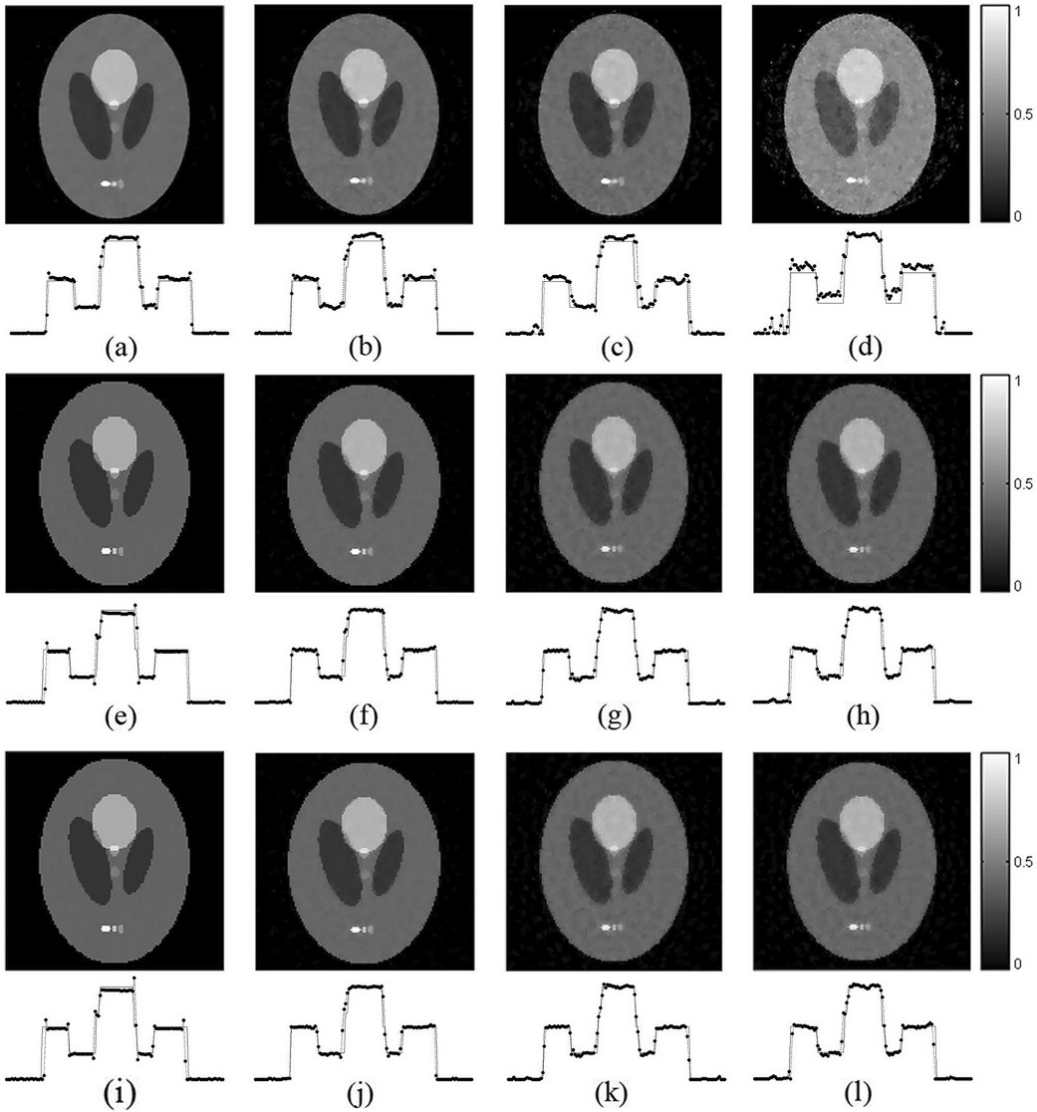
**The reconstruction results from noisy signal.** The first to the third columns show images reconstructed by the TV-GD **(a-d)** and TV-Lp algorithm with p = 0.8 **(e-h)** and p = 0.5 **(i-l)**, respectively, from signals with SNR of 10 dB, 5 dB, 3 dB and 0 dB.

The reconstruction results are shown in Figure 7. In the first row to the last row of the Figure 7, the signal to noise ratio (SNR) of the polluted measurements is 10 dB, 5 dB, 3 dB and 0 dB, respectively. As shown in the image, when the power level of the noises is not very strong (10 dB and 5 dB), the reconstructed images by using the noisy measurements have basically no obviously difference with the ones reconstructed with the noiseless signals. As the noise becomes stronger, the quality of the reconstructed images decreases.

We also plot the profiles of a pixel line in order to show the detail qualities of reconstructed images clearly. In Figure 7, the dotted line and the solid line are the pixel profiles of the reconstructed image and the original image, respectively. From the line plots, it is revealed that the proposed algorithm has better performance in the edge preservation and more accurate in the smooth area. We calculated the PSNR of the reconstructed image. The result is shown in Table 4. Our algorithm outperformed the TV-GD algorithm in any noise power level. Giving the credit to the optimal conditions, the reconstructed image is intended to be continuous and sharp. During the iteration, the photoacoustic signals get enhanced and the noise is suppressed.

**Table 4:** **PSNRs (dB) of the noise simulation image**

	10 dB	5 dB	3 dB	0 dB
TV-GD	32.24	28.01	22.44	16.96
TV-*L*_*p*_ (*p* = 0.8)	35.14	30.13	27.95	25.21
TV-*L*_*p*_ (*p* = 0.5)	35.63	30.40	28.10	25.06

As we can see from the table that the TV-*L*_*p*_ algorithm reconstructed images have a slightly better performance than the TV-GD algorithm when it comes to the image qualities. When the noise is extremely strong (0dB), the TV-*L*_*p*_ algorithm has a huge advantage than the TV-GD one in image quality. As for the different settings of parameter *p* in the TV-*L*_*p*_ algorithm, there is no major difference that can be observed between the two images. The PSNRs of the *p* = 0.5 setting is about 0.3 dB bigger than the *p* = 0.8 setting in the first three noise power level. But when the noise getting extremely strong (0 dB), the *p* = 0.8 setting is 0.1 dB bigger than the *p* = 0.5 setting in PSNR value.

From this part of simulation we can conclude that our TV-*L*_*p*_ algorithm is robust to noise and has a better performance than the TV-GD algorithm in the noisy measurement circumstance.

### Parameter investigation

As the original optimization problem of the image reconstruction is described in Eq. (14), the TV-*L*_*p*_ algorithm contains some parameters that are tunable, which are α, β, ϵ*, p* and ρ. In those parameters, the choice of ρ does not affect the performance of the TV-*L*_*p*_ algorithm theoretically. The result of the simulation also shows that the image quality is not sensitive to the parameter ρ for a large range. Here we set the parameter ρ to a steady value 1. The ϵ is the exiting condition parameter. It can be easily deduced that smaller ϵ will leads to slightly more accurate reconstructed image at the cost of more iteration times. In this part we focus on analyzing the parameter settings of *p*, α and β.

### Parameter setting of p

In the TV-*L*_*p*_ algorithm, we replace the *L*_1_ norm with the *L*_*p*_ norm (0 < *p* ≤ 1). It is reported in Ref. [35] that theoretically fewer measurements are required for accurate reconstruction in the *L*_*p*_ norm situation. But it also leads to failure in solving the optimization problem. It’s kind of a dilemma for the setting of *p*. So here we take different values of *p* to see its influence to the image reconstruction. The parameter α and β are both set to be 1 × 10^-2^.

We choose the 90-view and 18-view simulated photoacoustic signals that we used in ‘Sparse-view reconstruction’. We set the *p* value as 0.3, 0.5, 0.8 and 1. We calculate the reconstructed images’ PSNR value. It is shown in Table 5. When the *p* is set as 0.5, it has advantage in the quality of the reconstructed image. However, when the value of *p* continues to reduce to 0.3, there is no obvious improvement in image quality. But in the same time, the smaller the *p* is, the higher probability of the solving failure is during the simulation. The reduction of *p* leads to the increasing of the iteration times in our simulation. So taking these two factors into account, we set the *p* as 0.8 so that it can provide a great reconstruction performance and stability with a fast convergence.

**Table 5:** **PSNRs (dB) of reconstructed images with different** ***p***

	90-view	18-view
*p =* 0.3	39.26	36.62
*p* = 0.5	39.27	36.81
*p* = 0.8	39.05	36.72
*p* = 1	37.65	34.79

### Parameter settings of α and β

**Figure 8:**
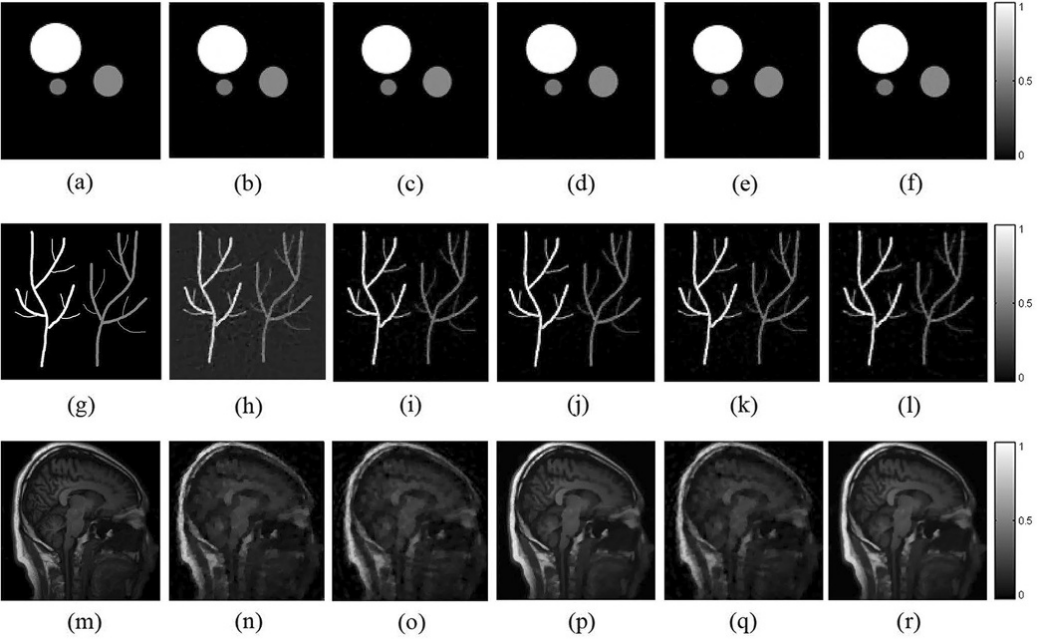
**The different image objectives reconstructed by TV-GD and TV-Lp algorithms.** The first to sixth rows refer to the original phantom **(a, g, m)**, the TV-GD reconstructed images **(b, h, n)**, the TV-Lp reconstructed images with parameter settings as (α = 1 × 10^-2^, β = 5 × 10^-3^) **(c, i, o)**, (α = 1 × 10^-2^, β = 1 × 10^-2^) (d, j, p), (α = 5 × 10^-2^, β = 5 × 10^-3^) **(e, k, q)** and (α = 5 × 10^-3^, β = 5 × 10^-3^) **(f, l, r)**, respectively.

As we describe above in Eq. (14), the parameter α and β are parameters corresponding to the weights of TV value and *L*_*p*_ norm value in this optimization problem respectively. We use these two parameters to balance the terms of the objective function. With different kinds of the objective image, the settings of those two parameters are different. Here we select three different images as the given optical energy deposition to test the universality of our algorithm and present further investigation of the parameter settings. We select a phantom that stand for the vessels and a phantom of dots with different energy degree in the simulation. We also choose a real brain MRI as the original optical energy deposition to demonstrate the performance of the proposed algorithm in reconstructing extremely detailed and complex structured imaging object. Here the TV-GD algorithm is used as a comparison. There are four groups of parameter settings, which are (α = 1 × 10^-2^, β = 5 × 10^-3^) , (α = 1 × 10^-2^, β = 1 × 10^-2^), (α = 5 × 10^-2^, β = 5 × 10^-3^) and (α = 5 × 10^-3^, β = 5 × 10^-3^). The reconstructed images are shown in Figure 8. From first row of Figure 6, we can see in the reconstruction of gradient sparse phantom. The TV based algorithm has great performance when the image demonstrate piecewise continuous behavior. All reconstructions are accurate and the background noise is suppressed well. When it comes to the images with the vessel phantom (Figure 8 (g)-(l)), those original optical energy depositions are a little bit more complex than the dots. The reconstruction results show that the image reconstructed by TV-*L*_*p*_ algorithm is better than the TV-GD ones. As in Figure 8(h), TV-GD image has some noises in the background and the edge of the vessel is blurred. While the TV-*L*_*p*_ images with different parameter settings both have high-resolution results. As for the real MRI image, it has very detailed information. As expected, both two groups of parameter setting α *=* β have the most accurate result among them. The increasing weighting of *L*_*p*_-norm condition can provide more detail information and prevent the reconstructed image emerging plantlike artifacts. The details such as edges and fine structures are well preserved in both reconstructions. The reconstruction results show that the TV-GD reconstructed image has severely paintinglike staircase artifacts with some loss in fine details. From our observation, TV-*L*_*p*_ algorithm with the parameter setting α *=* β preserves the fine features better than the TV-GD one. α and β are the regularization parameters determining the trade-off between the data consistency and the sparsity. It is revealed from the above simulation that the parameter setting α *=* β is a better strategy. In this parameter setting, the TV-*L*_*p*_ algorithm provides a 3 dB improvement in the PSNR over the TV-GD algorithm based on our calculation.

### Limited-view and irregular-view simulation

In the real application of PAI, due to the restrains of the shape or the size of the imaging object, a full angular scanning sometimes is hard to achieve. We evaluate the performance of the TV-*L*_*p*_ method in limited-view case, line-view case and un-equal-view case.

**Figure 9:**
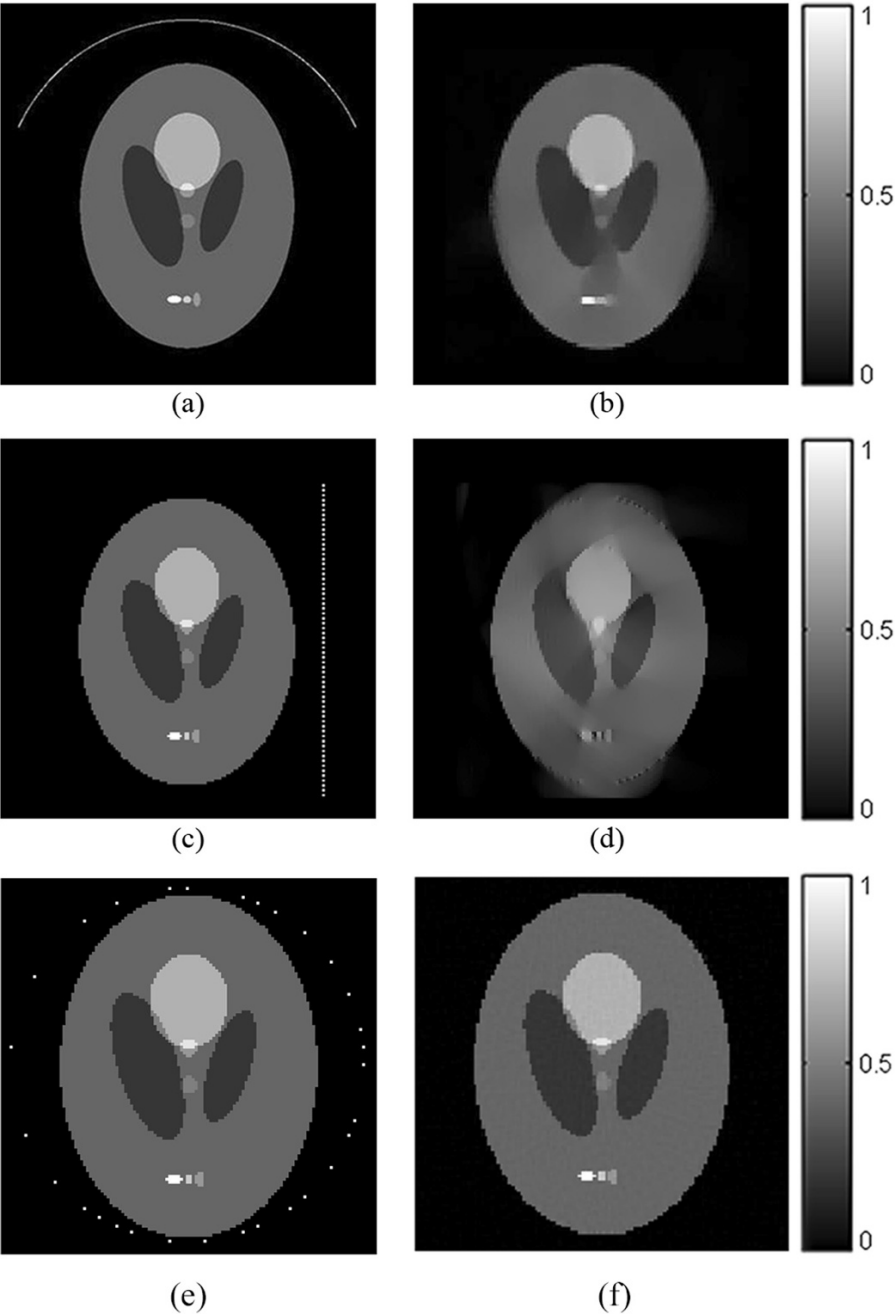
**Limited-view, line-view and un-equal angel step scanning reconstruction results. (a)** refers to the location of transducers in the limited-view simulation. **(b)** refers to the reconstructed image from the limited-view data. **(c)** refers to the location of transducers in the line-view simulation. **(d)** refers to the reconstructed image from the line-view data. **(e)** refers to the location of transducers in the un-equal angel step scanning. **(f)** refers to the reconstructed image from the un-equal angel step scanning data.

The simulation setup and the reconstructed image is shown in Figure 9. In the limited-view simulation (Figure 9(a)), the scanning angular range is set to 150° and the angular step is 3°, so 50-view photoacoustic signals are obtained. In the line-view simulation (Figure 9(c)), the transducer array with 60 transducers is placed in the right side of the imaging object and the interval between two transducer elements is 1.49 mm. It is revealed in Figure 9(b) and Figure 9(d) that the quality of the TV-*L*_*p*_ reconstruction is not much affected by the limitation of the sampling angle. Because the sampling angle is limited, the information definite is partly missed, yet the TV-*L*_*p*_ method can still provide a satisfying reconstruction. In un-equal angel step scanning, we randomly choose 30 sampling points from a 60-view projection and use these 30-view un-equal angle step data for image reconstruction. The result is shown in Figure 9(f). As we can see from the image, the reconstruction result can still maintain a very high quality.

## *In-vitro*experiments

### Experiment setup

We carry out the experiments on *in-vitro* signals to demonstrate the proposed TV-*L*_*p*_ algorithm’s performance in the practical application.

**Figure 10:**
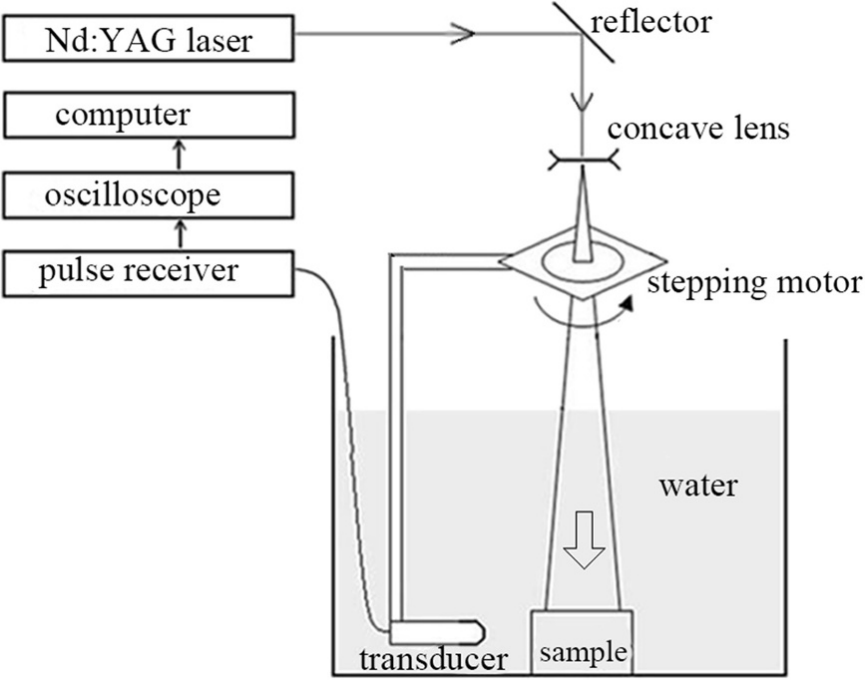
**The scheme of the experiment platform.**

The framework of the experiment platform is shown in Figure 10. In this platform, an Nd:YAG laser generator (Continum, Surelite I) is used to emit the laser pulse. The wavelength of the laser is 532 nm. A single laser pulse is generated at the frequency of 10 Hz and last 6-7 ns. The incident laser pulse is emitted towards the top of the phantom through a concave lens with the diameter of 5 cm. The setup of the lens enlarges the illumination area and lead to the pulse energy reduction in the illumination area. The energy is about 6.47 mJcm^-2^, which is lower than the ANSI laser radiation safety standard (20 mJcm^-2^) [1]. Signal acquisition is done by a water-immersion ultrasound transducer (Panametric, V383-SU). The transducer is a linearly unfocused one at 3.5 MHz (-6 dB bandwidth at 45%). A digital stepping motor (GCD-0301 M) is used to rotate the ultrasound transducer around the phantom placed in water. The scanning radius is 38 mm. The received analog ultrasound signals are amplified by a pulse receiver (Panametric, 5900PR). An oscilloscope (Agilent, 54622D) with the sampling frequency of 16.67 MHz is set to transform the received signals into digital ones. Both the laser generator and the digital motor are controlled by the computer through the serial interface. The transformed digital data is transported to the computer through the general purpose interface bus (GPIB).

**Figure 11:**
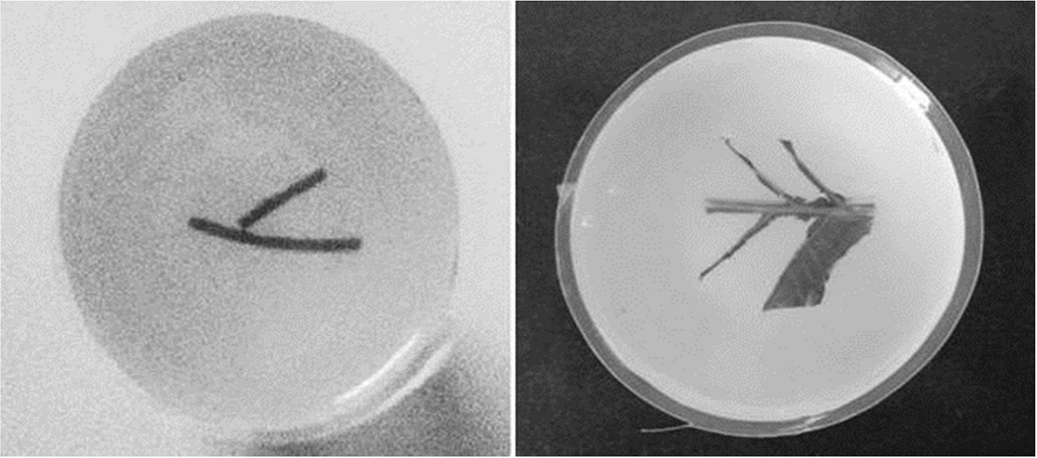
**The photos of the imaging samples.**

The imaged phantom we used in the experiment is made by gelatin cylinder. It is shown in Figure 11. There are two different phantoms. The radius of the phantom is 25 mm. The left one is made by two rubber bars with 1 mm diameter that embedded as the optical absorbers. The right one utilizes leaf which pretends as vein and tissue as the optical absorbers.

In the experiment, the transducer tends to measure the photoacoustic signal in-plane only, and the reconstruction is also in 2D. The cross-sectional image in any plane is mainly determined by the measured data in the same plane, and a set of circular measurement data on the same plane would be sufficient to reconstruct a good image. We use the deconvlution calculation before the reconstruction to eliminate the transducer’s impulse response influence.

### Experiment result

**Figure 12:**
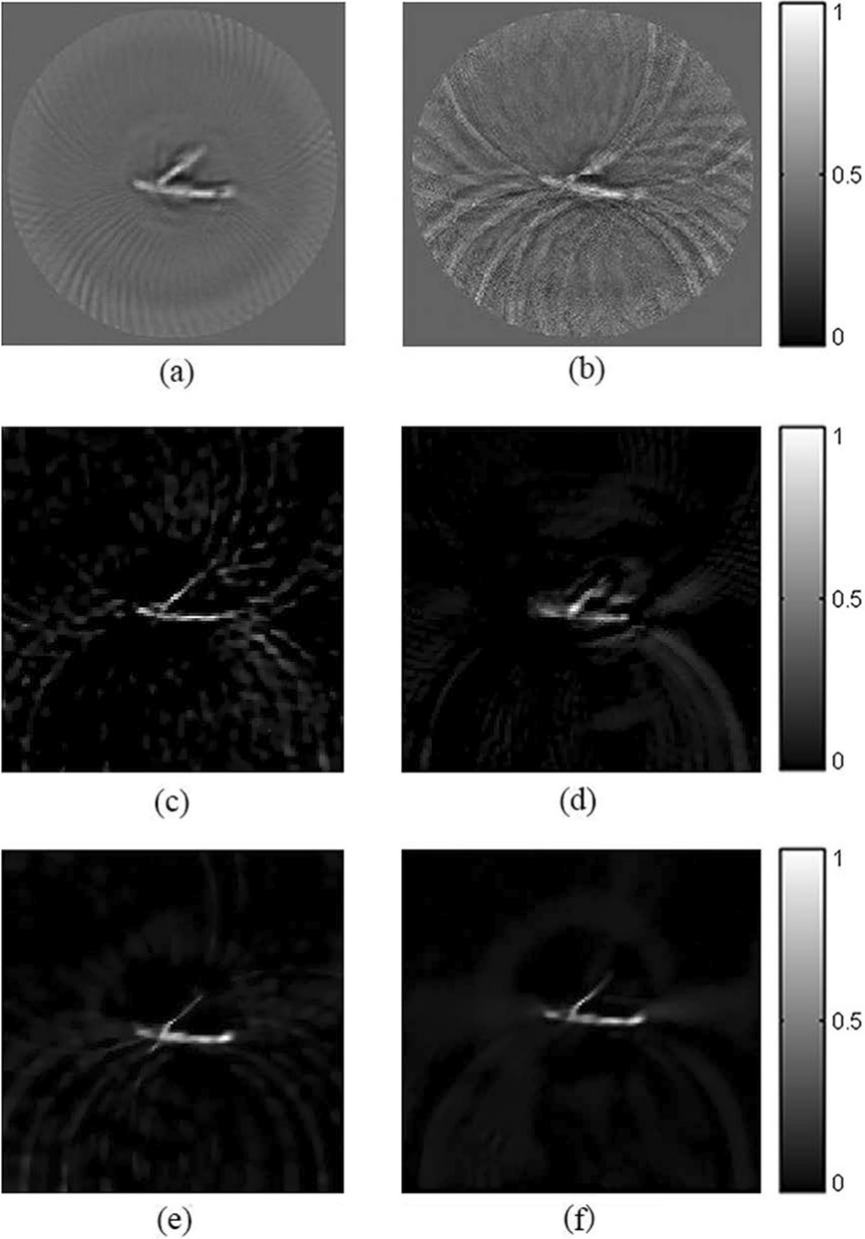
**The rubber phantom reconstruction results from experiment data.** From left to right, the reconstructed images of rubber sample from 90-view and 30-view experiment data respectively. The first to third rows refer to the images reconstructed by the FBP **(a, b)**, the TV-GD **(c, d)** and the TV-Lp **(e, f)** algorithm respectively.

In the experiment, 90-view and 30-view data are collected for reconstruction. The images are constructed by the FBP, the TV-GD and the TV-*L*_*p*_ algorithms, respectively. The reconstruction results are shown in Figure 12. The left column of the Figure 12 is reconstructed from 90-view data. When the sampling data is sufficient, all three algorithms are effective. With respect to the locations and sizes, the optical absorbers are all well reconstructed in the figure. While the FBP reconstructed image is not as clear as the images reconstructed by the iterative algorithms. When we reconstruct the image with a small of sampling angles (right column of Figure 11), the artifacts start to emerge in the FBP reconstructed image and the quality of the image is severely affected. But the TV-GD and the TV-*L*_*p*_ algorithms can still provide high-contrast images with less noise. In Figure 12(f), it is shown that the image reconstructed by the TV-*L*_*p*_ algorithm outperforms other algorithms in image contrast and noise suppression. The structure of optical absorbers is clear and the noise in the background is well-suppressed. The sparse-view of sampling has barely any influence on the quality of the TV-*L*_*p*_ reconstructed image.

**Figure 13:**
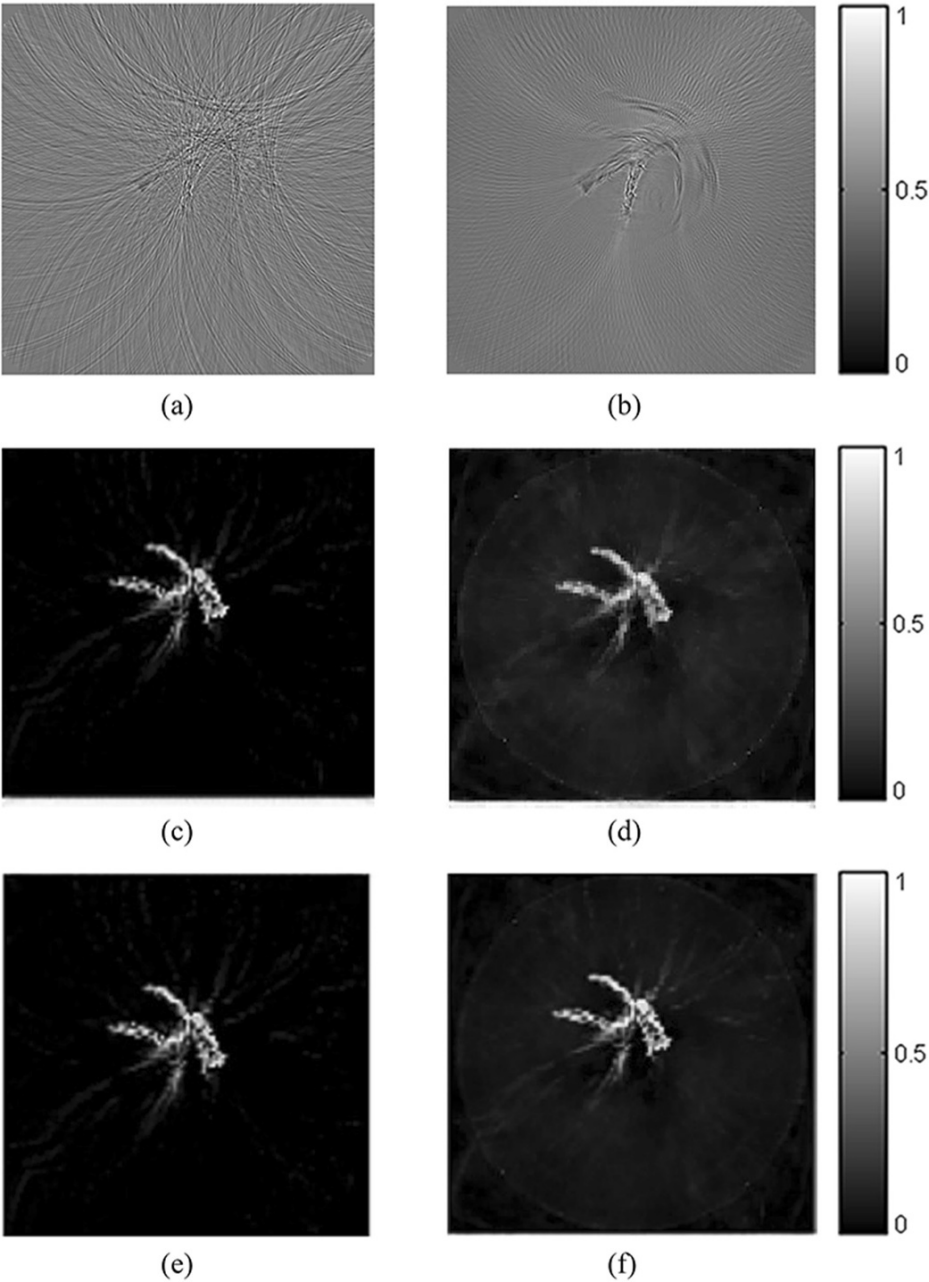
**The leaf vein phantom reconstruction results from experiment data.** From left to right, the reconstructed images of leaf vein phantom from 90-view and 30-view sampling experiment data respectively. The first to third rows refer to the images reconstructed by the FBP **(a, b)**, the TV-GD **(c, d)** and the TV-Lp **(e, f)** algorithm respectively.

*In vitro* imaging of a leaf vein is also performed to further demonstrate the advantages of the TV-*L*_*p*_ algorithm. The reconstruction result is shown in Figure 13. As the structure of the phantom is more complex, the FBP are deeply influenced by the artifact and fail to reconstruct the accurate image both under 90-view and 30-view sampling circumstance. It is shown in the figure, that the TV-GD and TV-*L*_*p*_ algorithms can still reconstruct the image in a high contrast level. But when the data is insufficient, there is some noise emerging in the background. TV-*L*_*p*_ algorithms can suppress the noise better than the TV-GD one. The optical absorber in TV-*L*_*p*_ one is more distinct than that in the image by TV-GD algorithm.

### Quantitative comparisons

**Figure 14:**
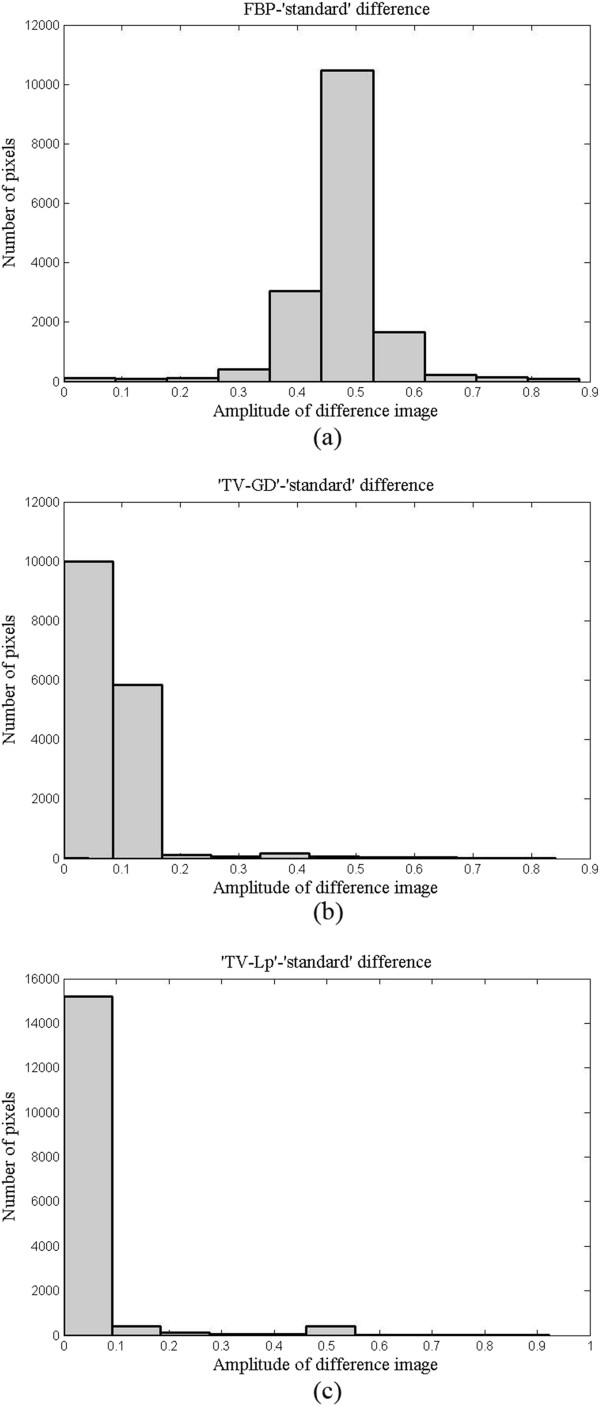
**The amplitudes histograms of the difference between reconstruction results and the “standard” one.** We use the image reconstructed by L1-norm with data from all 180 transducer elements as the standard. **(a)** Histograms image of the FBP algorithm. **(b)** Histograms image of the TV-GD algorithm. **(c)** Histograms image of the TV-Lp algorithm.

We use the *L*_1_-norm algorithm to reconstruct the image of 180-view data from the leaf vein phantom. As the sampling view is efficient, the reconstructed image is used as a “standard” one. We calculate the histograms of the difference between the reconstructed one and the “standard” one as shown in Figure 14. Figure 14 (a)-(c) are the difference histograms between the standard and the images reconstructed by the FBP, the TV-GD and the TV-*L*_*p*_, respectively, with 30-view data. In Figure 14, two CS-based algorithms have a large number of pixels with small ranges of difference with the standard one, which suggests that these two algorithms can reconstruct the image more accurately. In the case of the TV-*L*_*p*_ algorithm, the major part of the pixel difference is in the range from 0 to 0.1. From this experiment, the results demonstrate that the TV-*L*_*p*_ method can outperform the TV-GD one in the field of the image quality.

From the experiment result noted above, it is safe to say that the TV-*L*_*p*_ algorithm would have better performance in sparse-view PAI than other algorithms. It could provide stable and accurate reconstruction in both sufficient data sampling and sparse-view sampling situation.

## Conclusion

Aiming to reduce the scanning time and enhance the imaging quality of the photoacoustic image reconstruction, we proposed the TV-*L*_*p*_ algorithm that applies the total variation method and nonconvex optimization method to the PAI. The main idea of the algorithm is to apply *L*_*p*_-norm nonconvex optimization along with the total variation method. In the proposed algorithm, the Barzilai-Borwein step size selection method is adopted to provide faster convergence and smaller calculation. The effectiveness and universality of the algorithm is demonstrated through the numerical simulations. The numerical simulations show that the TV-*L*_*p*_ algorithm provides good imaging quality in sparse-view sampling situation. The algorithm convergence, the robustness to noise and the tunable parameters are also discussed. The simulation result reveals that the TV-*L*_*p*_ algorithm is a stable image reconstruction method with fast convergence and small computational cost. The TV-*L*_*p*_ algorithm is further investigated through some experiments using gelatin-made phantom. Compared with the result of other popular image reconstruction method, the TV-*L*_*p*_ imaging algorithm has significant advantage on contrast and noise suppression. From the discussion noted above, it could be concluded that the TV-*L*_*p*_ algorithm may be a practical algorithm for sparse-view photoacoustic imaging reconstruction.

## Abbreviations

PAI: Photoacoustic imaging
TV-*L*_*p*_: Total variation and *Lp*-norm
OAT: Optoacoustic tomography
TAT: Thermoacoustic tomography
FBP: Filtered back-projection
TV: Total variation
TVM: Total variation minization
ASD-POCS: Adaptive steepest-descent-projection onto convex sets
TV-GD: Total variation based gradient descent
MRI: Magnetic resonance imaging.
